# An improved dataset of force fields, electronic and physicochemical descriptors of metabolic substrates

**DOI:** 10.1038/s41597-024-03707-0

**Published:** 2024-08-27

**Authors:** Alessio Macorano, Angelica Mazzolari, Giuliano Malloci, Alessandro Pedretti, Giulio Vistoli, Silvia Gervasoni

**Affiliations:** 1 Dipartimento di Scienze Farmaceutiche, Università degli Studi di Milano, via Mangiagalli 25, 20133 Milano, Italy; 2 Dipartimento di Fisica, Università degli Studi di Cagliari, Cittadella Universitaria, S.P. Monserrato-Sestu Km 0.7, I-09042 Monserrato, CA Italy

**Keywords:** Computational chemistry, Cheminformatics, Combinatorial libraries

## Abstract

*In silico* prediction of xenobiotic metabolism is an important strategy to accelerate the drug discovery process, as candidate compounds often fail in clinical phases due to their poor pharmacokinetic profiles. Here we present Meta^QM^, a dataset of quantum-mechanical (QM) optimized metabolic substrates, including force field parameters, electronic and physicochemical properties. Meta^QM^ comprises 2054 metabolic substrates extracted from the MetaQSAR database. We provide QM-optimized geometries, General Amber Force Field (FF) parameters for all studied molecules, and an extended set of structural and physicochemical descriptors as calculated by DFT and PM7 methods. The generated data can be used in different types of analysis. FF parameters can be applied to perform classical molecular mechanics calculations as exemplified by the validating molecular dynamics simulations reported here. The calculated descriptors can represent input features for developing improved predictive models for metabolism and drug design, as exemplified in this work. Finally, the QM-optimized molecular structures are valuable starting points for both ligand- and structure-based analyses such as pharmacophore mapping and docking simulations.

## Background & Summary

The prediction of drug metabolism has been attracting great interest in recent years for its capacity to rapidly screen huge databases of compounds allowing a cost-effective discarding of the molecules with a predicted unfavourable profile. Notably, such an *in silico* screening can be performed in the early phases of the drug discovery process with clear benefits in the reduction of the failures related to pharmacokinetic and toxicological concerns^1^.

The approaches for metabolism prediction can be subdivided into two major groups. On one hand, the local methods focus on a specific metabolic reaction and on the related metabolizing enzyme(s). On the other hand, global methods aim to predict the overall metabolic fate a given compound can undergo. Even though the global approaches often involve knowledge-based metabolic rules, local and global methods can develop their predictive models by exploiting both ligand- and structure-based approaches^2^. Over the last years, all metabolism predictive studies greatly benefit from the artificial intelligence algorithms which allow the predictive performances to be constantly enhanced^3^.

The major factor which has so far limited the development of metabolism predictive models (especially involving global methods) is the scarcity of highly accurate and extended datasets. Most available metabolic resources are indeed collected by automatic interrogation of other databases^4^ combining xenobiotic and endogenous metabolic data for omics analyses^5^. Hence, we recently proposed the MetaQSAR resource^6^, a manually curated database collected by meta-analysis of the recent primary specialized literature. MetaQSAR comprises 3788 first generation metabolic reactions which are grouped by a finely organized classification which subdivides them in 3 major classes, 21 classes and 101 subclasses^7^. MetaQSAR is thus a fruitful source of highly accurate datasets well suited for developing metabolism predictive analyses which indeed proved successful in both local^3^ and global ligand-based studies^8,9^. Altogether, the developed predictive models emphasized the key role of electronic descriptors, a quite expected outcome when considering their capacity to parameterize the intrinsic reactivity of each atom/molecule. The hitherto published studies involved electronic descriptors as computed by semi-empirical methods, an almost compulsory choice to reduce the computational costs^10^. Nevertheless, one may imagine that the predictive power of these descriptors should parallel the level of theory by which they are calculated.

Hence, we undertook a highly demanding campaign of DFT calculations in which all the 2054 first generation substrates, as extracted from MetaQSAR, underwent DFT-based full optimization and frequency calculations. Here, we release all the so derived molecular data for more than 2000 molecules including: (a) two datasets of all the QM-optimized substrates (at both DFT and semiempirical PM7 levels, with the corresponding Gaussian output files); (b) an homogeneous database of General Amber Force-Field parameters including several compounds bearing non-standard atoms; (c) all the derived electronic descriptors; (d) an extended set of physicochemical descriptors as computed by using the DFT-optimized conformations.

By considering the structural richness of the simulated molecules, the present data can have many applications. First, the collected force-field parameters can be used to perform molecular mechanics calculations as exemplified by the here reported validating molecular dynamics runs on the compounds including non-standard atoms. Second, as exemplified by few selected test-cases, the computed descriptors can be utilized to develop improved predictive models (not necessarily focused on drug metabolism). Third, the QM-optimized structures can represent valuable starting points for various ligand- and structure-based studies. Notice that the collected dataset of optimized structures mostly comprises marketed drugs and drug-like molecules and, therefore, it can be particularly suited for repurposing and virtual screening campaigns.

## Methods

### DFT-optimization of MetaQSAR molecules

**Fig. 1:**
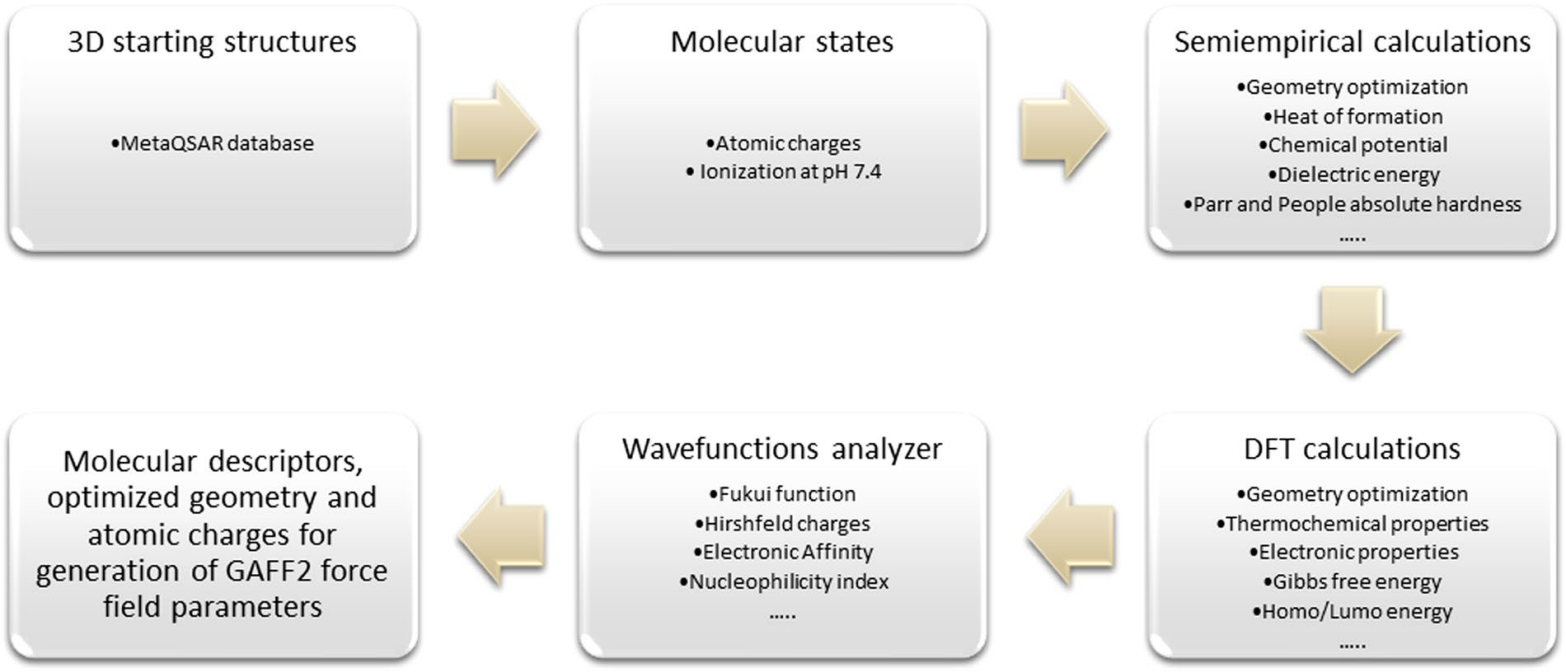
Schematic view of the computational workflow adopted in this work.

As schematized in the workflow of Fig. 1, the 3D structures of the first-generation substrates contained in the MetaQSAR database^7^ (overall 2054 molecules) were generated at physiological pH 7.4 by the VEGA^11^ program.

All compounds underwent a two steps geometry optimization, using first a semi-empirical and then a Density Functional Theory (DFT)^12^ level of theory. In detail, the semi-empirical calculations were performed using the MOPAC 2016 software^13^ and the PM7^14^ Hamiltonian. The DFT calculations included full optimization and frequency calculation using the Gaussian 16 software (Revision A.03)^15^. The hybrid B3LYP functional^16^ is widely recognized as the standard for the systematic study of organic molecules^17,18^. It has been used in combination with the 6–31 G* basis set for C and H, and 6–31 + G* for heteroatoms such as N, O, P, S. For compounds containing “non-standard” atoms (*i.e*., Pt, As, Hg, Se, Pb), LANL2DZ^19^ effective core potential (ECP) and double zeta basis set were used. In all cases, the absence of imaginary frequency modes for the optimized structure of the ligand confirms a true minimum on the potential energy surface. At the optimized geometry, the Multiwfn 3.8 program^20^ was used to calculate Hirshfeld Charges^21^ within the conceptual density functional theory (CDFT)^22^. Hirshfeld population analysis (HPA) has proven to be a suitable choice compared to other population analysis schemes^23,24^. It is particularly effective for studying and obtaining Fukui functions^25^, dual descriptors and Hirshfeld charges^21^ itself, which reveals nucleophile and/or electrophile reactive centers of the ligand that underwent a metabolic reaction. GuassSum^26^ 3.0 was used to extract all the information associated with each molecular orbital, from the previously generated output files.

To check the quality of DFT calculations, we compared the DFT optimized structures with experimental crystallographic structures retrieved from the Cambridge Structural Database (CSD)^27^ of the Cambridge Crystallographic Data Center (CCDC). In detail, a subset of 100 molecules were selected from the MetaQSAR database considering their structural diversity, by means of RDkit diversity picker^28^ as implemented in the KNIME 4.6.4 analytic platform^29^. We restricted the selection to CSD experimental structures with R% factor value < 5. The root mean square deviation (RMSD) values on heavy atoms, between DFT optimized structures and experimental structures were calculated by using the Visual Molecular Dynamics software (VMD)^30^ along with the corresponding RMSD_w_ (average value) (see Technical Validation section).

### Amber force-field parameters generation

General Amber Force-Field parameters (GAFF2)^31^ were generated starting from the Gaussian log files and assigning the Hirshfeld atomic charges obtained as described above. For compounds containing non-standard atoms (*i.e*., selenium. platinum, arsenic, iron, silicon, mercury, tin, and boron) we generated bonded parameters following the metal center parameter builder procedure (MCPB.py)^32^ as implemented in Amber22^33^. For compounds containing boron atoms, not supported by the MCPB.py procedure, we used the parameters reported by Tafi *et al*.^34^. For molecules containing non-standard atoms the quality of the GAFF2 parameters was checked by performing a molecular mechanics optimization using the conjugated gradient algorithm followed by a 100 ns-long molecular dynamics simulation in explicit water solution using Amber22. In detail, compounds were inserted into a box of OPC water molecules^35^ and the systems were neutralized by adding either Na^+^ or Cl^−^ counter ions. The hydrogen mass repartition scheme was adopted^36^, as well as the SHAKE algorithm^37^. The NPT production runs were preceded by an energy minimization, a heating followed by a cooling phase, as described previously^38^. We used a time step of 4 fs, a cutoff for non-bonded interaction of 9 Å, the Langevin thermostat and the Berendsen barostat for keeping the temperature at 310 K and the pressure at 1 Atm. Periodic boundary conditions and PME method were applied.

### Metabolism prediction model building

To show how the molecular descriptors provided by our study can be helpful in predicting the metabolism of compounds, we built machine learning models to predict whether a compound undergoes three selected metabolic reactions: glutathione or generic sulfur conjugation (MetaQSAR class 24), hydrolysis of amides, lactams, and peptides (MetaQSAR class 12), and oxidation and reduction of sulfur atoms (MetaQSAR class 08). A binary classification model based on the MetaQSAR system was used. The program Weka 3.8.6^39^ was used to build the model, using the Random Forest algorithm with the following parameters: (1) batch size = 100; (2) number of threads = 1; (3) number of iterations = 100; (4) the attribute importance was not evaluated. The most significant features were selected by using the Weka program according to both the BestFirst search algorithm (direction = Forward; lookupCacheSize = 1; searchTermination = 5) and theWrapperSubsetEval attribute evaluator (classifier = RandomForest with default settings; doNotCheckCapabilities = False; evaluationMeasure = accuracy,RMSE; folds = 5; seed = 1; threshold = 0.01). The performance of the models was evaluated using different metrics: Precision and Recall, see Eqs. (1–5)), Matthew’s Correlation Coefficient (MCC), and the Receiver Operating Characteristic Curve Area (ROC Area)^40^, obtained through a 10-fold cross-validation. Specifically, in the following equations, each symbol is represented as follows: TP for true positive, TN for true negative, FP for false positive, and FN for false negative.

1 $${\rm{MCC}}:\frac{{TP}\times {TN}-{FP}\times {FN}}{\sqrt{\left({TP}+{FP}\right)\left({TP}+{FN}\right)\left({TN}+{FP}\right)\left({TN}+{FN}\right)}}$$

2 $${\rm{Precision\; Class\_YES}}:\frac{{TP}}{{TP}+{FP}}$$

3 $${\rm{Precision\; Class\_NO}}:\frac{{TN}}{{TN}+{FN}}$$

4 $${\rm{Recall\; Class\_YES}}:\frac{{TP}}{{TP}+{FN}}$$

5 $${\rm{Recall\; Class\_NO}}:\frac{{TN}}{{TN}+{FP}}$$

## Data Records

**Fig. 2:**
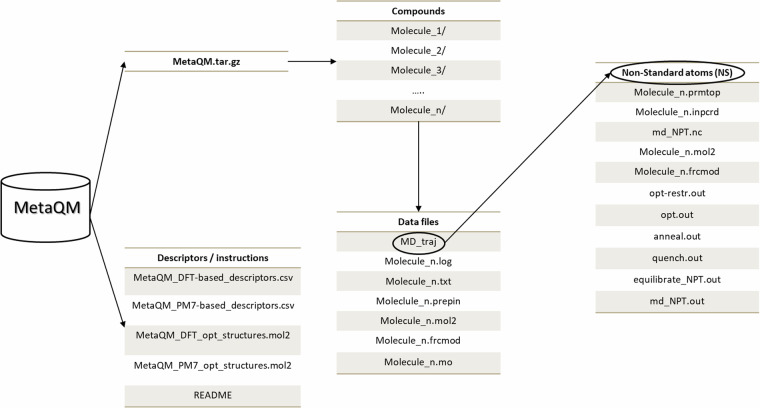
Schematic representation of Meta^QM^ structure.

The Meta^QM^ database is available on figshare^41^. Figure 2 shows the structure of the database.

We shared two comma separated files containing molecular descriptors as derived from semiempirical optimized structures (*MetaQM_PM7-based_descriptors.csv*) and from DFT optimized structures (*MetaQM_DFT-based_descriptors.csv*). The list of computed descriptors together with a precise description of their meaning is reported in Supporting Information (Table S1). The DFT and PM7 optimized structures are contained in two MOL2 database files (*MetaQM-DFT_opt_structures.mol2* and *MetaQM-PM7_opt_structures.mol2*).

The compress file *MetaQM.tar.gz* contains 2054 folders, one for each compound; the folder and the included files are named after the compound (*e.g*., Abacavir/). Each directory includes 6 files: the Gaussian output of DFT calculations (*e.g*., *Abacavir.log*), the list of atomic charges computed at DFT-level of theory (*e.g*., *Abacavir.txt*), the list of all molecular orbitals (*e.g*., *Abacavir.mo*), the .mol2 file used for the force field generation (*e.g*., *Abacavir.mol2*), and the two GAFF2 files (*e.g*., *Abacavir.prepin*, *Abacavir.frcmod*). For compounds containing non-standard atoms (Table S2) the .lib file is supplied instead of .prepin (*e.g*., *Arsenate.lib*). For the Ferroquine compound, only the .log and .txt files are supplied (see Methods).

For compounds with non-standard atoms an additional subfolder (MD_traj/) is further provided. MD_traj/ contains the topology and coordinates (*e.g*., *Arsenate.prmtop*, *Arsenate.inpcrd*) of the solvated compound used for the validating MD simulation. The MD trajectory is contained in the md_*NPT.nc* file. The Amber22 output files from minimization, equilibration and production steps are supplied as well *(opt-restr.out*, *opt.out*, *anneal.out*, *quench.out*, *equilibrate_NPT.out*, *md_NPT.out*).

## Technical Validation

### DFT-optimized structures

The validation of molecular structures optimized by DFT calculations was carried out by selecting 100 structurally diverse molecules from the simulated MetaQSAR substrates. We restricted the selection to the experimental crystal structures deposited on CSD with high quality resolution (*i.e*., R% < 5). In the case of compounds with more available structures, we chose the one with the lowest R% value.

The resolved structures of the so selected 100 compounds were then compared with the corresponding DFT optimized conformations. For each selected compound, Table S3 compiles the reference CSD code and R-factor together with the resulting RMSD value. The RMSD mean value (RMSD_w_) is also reported.

Almost 70% of the molecules have RMSD values < 1 Å, indicating that the DFT optimized structures are in good agreement with the corresponding resolved structures, with the lowest value being 0.01 Å for Tetrafluoroethene, Coumarin and Dioxane. In contrast, the 9% of the cases show large structural difference with RMSD > 2 Å, the maximum value of 3.49 Å being observed for Dabrafenib. As expected, the obtained results suggest that flexible molecules give rise to high RMSD values, while rigid molecules reveal low RMSD values. However, the RMSD mean value of 0.76 Å confirms an overall agreement between the DFT optimized conformations and the experimental structures.

The molecular descriptors were computed on the DFT optimized conformations, that can differ in the general case from the conformations of compounds when in complex with metabolic enzymes. Therefore, to check the robustness of the provided dataset, we collected a subset of 20 diverse representative compounds (ranging from 12 to 50 heavy atoms, and 0 to 18 flexible torsions), for which the experimental structures in complex with metabolic enzymes are available in the Protein Data Bank^42^. We then computed the QM-based descriptors (with both DFT and PM7 methods) on the experimental conformation, and we compared the results with those derived from the corresponding QM-optimized geometries (Supporting_TableS 4a, Supporting_TableS 4b). We obtained small differences between the different series of descriptors, with average percentage variations of 11% for the PM7-based descriptors, and 6% for DFT-based descriptors, indicating the overall reliability of the data.

### Amber force-field parameters

To validate the quality of the GAFF2 parameterization for molecules containing non-standard atoms, we compared the optimized geometries derived from the molecular mechanics minimization with those obtained by DFT calculations. Table 1 shows the RMSD values between the two structures, computed on all atoms. In 9 cases out of 16, the two compared structures are almost identical (*i.e*., RMSD < 0.5 Å) and only one molecule shows a RMSD value greater than 1 Å. Overall, the average value considering all “non-standard” cases is equal to 0.49 Å thus confirming the reliability of the computed force field parameters. For the same molecules, the force field parameters were utilized to perform 100 ns-long MD simulations in explicit water solution to further test the reliability of the bonded parameters. The visual inspection of the MD trajectories, available on figshare, reveals a satisfactory stability of distances/angles/torsions involving non-standard atoms along the 100 ns timescale, thus demonstrating the reliability of the corresponding bonded parameters.

**Table 1:** RMSD values (Å) as computed on all atoms between molecular mechanics and DFT-optimized structure of compounds containing non-standard atoms.

Compound	RMSD (Å)	Formula	Molecular weight	Atoms
Ar-67	0.76	C_26_H_30_N_2_O_5_Si	478.61	64
Arsenate	0.44	HO_4_As	139.93	6
ARSENITE	0.61	H_3_O_3_As	125.94	7
Bortezomib	0.83	C_19_H_25_N_4_O_4_B	384.24	53
Carboplatin	0.47	H_6_N_2_Cl_2_Pt	300.04	11
Cisplatin	0.41	C_2_H_7_O_2_As	137.99	12
Dimethyl arsinate	0.76	C_2_H_6_Sn	148.78	9
Dimethyltin	0.40	C_11_H_17_NO_4_B	238.07	34
GSK2251052	1.13	C_12_H_15_N_6_OS_2_As	398.34	37
Melarsoprol	0.76	Cl_2_Hg	271.50	3
Mercury chloride	0.06	CH_3_Hg	215.62	5
Methylmercury	0.01	CH_3_ClHg	251.08	6
Methylmercury chloride	0.03	C_8_H_14_N_2_O_4_Pt	397.29	29
Oxaliplatin	0.05	C_5_H_11_NO_2_Se	196.11	20
Seleno-L-methionine	0.50	C_20_H_24_NO_2_FClSi	392.95	50
Sila-Haloperidol	0.56	C_6_H_12_N_2_O_4_Pt	371.25	25
Average	**0.49**			

### Example of metabolic predictions using Meta^QM^

To test how the Meta^QM^ descriptors can feed predictive machine learning models of metabolism, we performed three tests of selected metabolic predictions. Specifically, we followed the MetaQSAR metabolic reaction classification system to predict whether compounds undergo: (1) glutathione conjugation (metabolic class 24, 169 substrates plus 169 non-substrates), (2) hydrolysis (metabolic class 12, 117 substrates plus 117 non-substrates), and (3) oxidation and reduction of sulphur atoms (metabolic class 08, 127 substrates plus 127 non-substrates). For each prediction model, we used a balanced data set consisting of 50% of molecules that undergo the reaction (substrates) and 50% of molecules that do not undergo the reaction (non-substrates). To highlight the role of electronic descriptors, each reactive functional group was also used for non-substrate species. The results of the prediction models are shown in Table 2.

**Table 2:** Performances of the three machine-learning metabolic predictions based on Meta^QM^ descriptors.

	n	MCC	ROC Area	Precision YES	Precision NO	Recall YES	Recall NO
Class 24	338	0.71	0.92	0.87	0.84	0.83	0.88
Class 12	234	0.41	0.75	0.73	0.68	0.64	0.77
Class 08	254	0.68	0.89	0.85	0.83	0.82	0.86

The MCC value ranges from −1 (worse) to 1 (best), all the other metrics range from 0 (worse) to 1 (best). The overall number of instances for each model is also reported (n).

Overall, the performance of the three prediction models is satisfactory, with the glutathione conjugation reaction (class 24) and oxidations of sulphur atoms (class 08) showing the best results with an MCC of 0.71 and 0.68, respectively. The ROC curves (*i.e*., true positive rate (TPR) vs False Positive Rate (FPR)) for all the three classes are reported in Figure S1. Class 12, representing the hydrolysis of amides, lactams and peptides, obtained a lower but acceptable performance in terms of prediction. These results could be related to the MetaQSAR classification scheme of reactions, for which both conjugation reactions and oxidation on sulphur atoms (classes 24 and 08) include more homogeneous metabolic reactions. Instead, for class 12 (hydrolysis of amides, lactams and peptides), the collected metabolic reactions are more heterogeneous, which may partly explain the lower but acceptable performance of the corresponding model. Although the samples used to build each model included the same reactive functional group for both substrates and non-substrates, that can make the prediction more difficult, the novel electronic descriptors presented here show an overall satisfactory performance.

All predictive models contain three types of molecular descriptors (*i.e*., phys-chem, DFT-based, and semiempirical) (Table 3) obtained after 10-fold cross validation (Table 3).

**Table 3:** Features selection for the three test-case metabolic predictive models (see Table S1 for details about each molecular descriptor).

Class 24	Class 12	Class 08
ChiralAtms	EzBnds	Mass
HbDon	ChiralAtms	HbDon
Impropers	Psa	ChiralAtms
Rings	Impropers	Psa
**Hirshfeld_positive_charges**	VirtualLogP	**Gap**
**piS_TOTAL**	**Hirshfeld_positive_charges**	**Chemical_potential**
	**Fukui_positive**	**Ionization_potential**
	**D.E_Total_PM7**	**Nucleophilicity_index**
		**Thermal_energy**
		**Fukui_negative**

QM-based electronic properties are highlighted in bold.

Class 08 is characterized by the highest number of features with respect to the other two classes, especially considering the electronic parameters. This could be ascribed to the complex biochemical mechanisms of the oxidation reaction catalyzed by CYP450 on the sulfur atom. In detail, the Cdp I complex is also referred to as an “electrophilic oxidant”^43^, which could explain why both *Fukui_negative* and *Nucleophilicity_Index*, both capturing atomic and molecular nucleophilicity, are identified as important features for this model. In addition, other electronic descriptors are identified, that globally describe the chemical reactivity of the oxidation reaction. The physicochemical parameters are related to molecular size and shape except for *HbDon* and *Psa*, which encode both polarity and the presence of chemical groups susceptible to metabolism. When considering the other two classes, the number of electronic features is lower, possibly due to the simpler reaction mechanisms compared to the previous one. In these cases, electronic parameters that encode both electrophilicity and chemical reactivity (*Hirshfeld_positive_charges*, *Fukui_positive*, *D.E_Total_PM7*, and *piS_TOTAL*) are found to be important, as well as physicochemical parameters accounting for molecular size, molecular shape and polarity/lipophilicity properties.

### Supplementary information

Supplementary Information

## Data Availability

The starting 3D structures of compounds were retrieved from the MetaQSAR^7^ database (available under licence). The ionization of compounds was performed using VEGA 3.2.1^11^. The software MOPAC^13^ 2016 was used for the semiempirical optimizations and for the calculation of semiempirical descriptors. Gaussian16^15^ (Revision A.03) was employed for both the DFT-based geometry optimizations and the descriptors collection, and Multiwfn 3.8^20^ was used only for the DFT-based descriptor computation. For each compound, GaussSum 3.0^26^ was used to extract the molecular orbitals from the Gaussian16 output files in combination with a personalized script (*extract_orbitals.py*). VMD 1.9.4^30^ was used for the visualization and computation of RMSD of compounds. Amber22^33^ was used for the generation of the force field parameters and the MD simulations. Weka 3.8.6^39^ was employed to create the metabolism predictive machine learning models.
