# Supplementary material for: An improved dataset of force fields, electronic and physicochemical descriptors of metabolic substrates
Source: Sci Data. 2024 Aug 27;11:929. doi: 10.1038/s41597-024-03707-0 (PMC11349763; doi:10.1038/s41597-024-03707-0)
Supplement: Supplementary file 1 — Supplementary Information [file 41597_2024_3707_MOESM1_ESM.pdf]

# *An improved dataset of force fields, electronic and physicochemical descriptors of metabolic substrates*

Alessio Macorano[1], Angelica Mazzolari[1], Giuliano Malloci[2], Alessandro Pedretti[1], Giulio Vistoli[1], Silvia Gervasoni*[2]

**Affiliations**
1. Dipartimento di Scienze Farmaceutiche, Università degli Studi di Milano, via Mangiagalli 25, 20133, Milano, Italy
2. Dipartimento di Fisica, Università degli Studi di Cagliari, Cittadella Universitaria, S.P. Monserrato-Sestu Km 0.7, I-09042 Monserrato (CA), Italy

corresponding author: Silvia Gervasoni (silvia.gervasoni@dsf.unica.it)

# SUPPORTING INFORMATION

| Feature (units) | Description |
|---|---|
| **PM7-based** | |
| HEAT_OF_FORMATION (kcal/mol) | Enthalpy variation, when one mole of a system is formed from its elements |
| DIELECTRIC_ENERGY (eV) | Stabilization energy from the interaction of the charges in the solute with the induced charges on the solvent accessible surface plus the electrostatic energy |
| ELECTRIC_DIPOLE_PM7 (D) | Product between absolute charge and distance between centre of positive and centre of negative charge |
| IONIZATION_POTENTIAL_PM7 (eV) | Minimum energy required to eject an electron out of a neutral atom or molecule in its ground state |
| MOLECULAR_WEIGHT (g/mol) | Mass of a given substance divided by the amount of a substance, defined as mol |
| COSMO_AREA ($Å^2$) | Surface of the molecule that can be reached by the by the center of charge of a solvent molecule |
| COSMO_VOLUME ($Å^3$) | Volume included in the COSMO surface |
| CHARGE_ON_SYSTEM | Net charge of the molecule |
| MULLIKEN_ELECTRONEGATIVITY (eV) | $-\alpha$ |
| PARR_&_POPLE_ABSOLUTE_HARDNESS (eV) | $\eta = ½ (\varepsilon_{homo} - \varepsilon_{lumo})$ |
| SCHUURMANN_MO_SHIFT_ALPHA (eV) | $\alpha = ½ (\varepsilon_{homo} + \varepsilon_{lumo})$, average of the Homo and Lumo energies |
| EHOMO_PM7 (eV) | Energy of the highest occupied molecular orbital |
| ELUMO_PM7 (eV) | Energy of the lowest unoccupied molecular orbital |
| Dn_TOTAL_PM7 ($eV^{-1}$) | Nucleophilic delocalizabilities, Total sums of all $D^N(r)$ (nucleophilic delocalizabilities) for each reactive center (r) of the molecule. It's based on the molecular orbital expansion coefficients, also as a measure of energy stabilization due to nucleophilic attack |
| De_TOTAL_PM7 ($eV^{-1}$) | Electrophilic delocalizabilities, Total sums of all $D^E(r)$ (electrophilic delocalizabilities) for each reactive centre (r) of a molecule. It’s based on the molecular orbital expansion coefficients, also as a measure for the energy stabilization due to electrophilic attack. |
| piS_TOTAL ($charge^2$/energy) | Self-polarizability $\pi S$ of an atom |
| **DFT-based** | |
| D.E Total_DFT ($Hartree^{-1}$) | Electrophilic delocalizabilities, defined as above, based on Hirshfeld partition method |
| D.N Total_DFT ($Hartree^{-1}$) | Nucleophilic delocalizabilities, defined as above, based on Hirshfeld partition method |
| VdW_volume ($Å^3$) | Molecular Volume based on vdW radii |
| Ehomo_DFT (eV) | Energy of the highest occupied molecular orbital |
| Elumo_DFT (eV) | Energy of the lowest unoccupied molecular orbital |
| Gap (eV) | Difference between homo and lumo energy |
| dipole_DFT (D) | Product between absolute charge and distance between centre of positive and centre of negative charge |
| Chemical_potential (eV) | Energy change of a system with respect to electron number at a fixed external potential |
| Electron_affinity_DFT (eV) | Energy change when an electron is added to a neutral molecule |
| Mulliken_electronegativity_DFT (eV) | Measure of resistance to the loss of electron density |

| | |
|---|---|
| Ionization_potential_DFT (eV) | Minimum energy required to eject an electron out of a neutral atom or molecule in its ground state |
| Hardness_DFT (eV) | Change of the electronic chemical potential respect to the electron number at a fixed external potential |
| Softness (eV) | Inverse of the chemical hardness |
| Electrophilicity_index (eV) | Electrophilicity of a molecule, measure of the energy stabilisation of a molecule when it acquires an additional amount of electron density from the environment |
| Nucleophilicity_index (eV) | Nucleophilicity of a molecule, expressed as energy difference between homo of nucleophile and homo of tetracyanoethylene |
| ThermalEnergy (kcal/mol) | Sum of electronic and thermal energies |
| Electronic_ZPE (kcal/mol) | Sum of electronic and zero-point vibrational energy |
| Enthalpy (kcal/mol) | Sum of electronic and thermal enthalpies |
| Gibbs_energy (kcal/mol) | Sum of electronic and thermal free energies |
| Hirshfeld_positive_charges (e) | Most positive value of atomic Hirshfeld charge |
| Fukui_positive (e) | Most positive value of positive Fukui function (Nucleophilic attack) |
| CDD_positive (e) | Most positive value of CDD (Difference between positive and negative Fukui function) |
| Hirshfeld_negative_charges (e) | Most negative value of atomic Hirshfeld charge |
| Fukui_negative (e) | Most positive value of negative Fukui function (Electrophilic attack) |
| CDD_negative (e) | Most negative value of CDD (Difference between positive and negative Fukui function) |
| **Physicochemical descriptors** | |
| Angles | Number of angles |
| Atoms | Number of atoms |
| Bonds | Number of bonds |
| Charge (e) | Total charge |
| ChiralAtms | Number of chiral atoms |
| Dipole | Product between absolute charge and distance between centre of positive and centre of negative charge |
| EzBnds | Number of bonds with E/Z geometry |
| FlexTorsions | Number of rotable bonds |
| Gyrrad (Å) | Radius of gyration. Measure of the distribution of atoms in a molecular structure with respect to either its center of mass or a given axis of rotation |
| HbAcc | Number of H-bond acceptor atoms |
| HbDon | Number of H-bond donor atoms |
| HeavyAtoms | Number of heavy atoms |
| Impropers | Number of improper angles |
| Lipole | Lipophilicity moment, measure of the lipophilic distribution in a 3D space |
| Mass (Da) | Molecular weight |
| MassMI | Monoisotopic mass |
| Ovality | Ratio Area/Volume, measure of how close the shape of a molecule is to a sphere or a cigar shape |

| | |
|---|---|
| Psa (Å$^2$) | Polar surface area, is defined as the amount of molecular surface arising from polar atoms (nitrogen and oxygen atoms together with their attached hydrogen atoms |
| Rings | Number of rings in the molecule |
| Sas (Å$^2$) | Solvent accessible surface |
| Sav (Å$^3$) | Solvent accessible volume |
| Sdiam (Å) | Surface diameter, diameter of the equivalent sphere with the same surface of the molecule |
| Surface (Å$^2$) | Molecular surface |
| Torsions | Number of torsion angles |
| Vdiam (Å) | Volume diameter, diameter of the equivalent sphere with the same volume of the molecule |
| VirtualLogP | Lipophilicity measure |
| Volume (Å$^3$) | Molecular volume |

**Table S1.** List of both physicochemical and electronic molecular descriptors calculated at the semiempirical PM7 and DFT level, with the corresponding description.

| Compound names | Non-standard atom |
|---|---|
| ar-67 | As |
| Arsenate | As |
| Arsenite | As |
| Bortezomib | B |
| Carboplatin | Pt |
| Cisplatin | Pt |
| Dimethyl_arsinate | As |
| Dimethyltin | Sn |
| Ferroquine | Fe |
| GSK2251052 | B |
| Melarsoprol | As |
| Mercury_chloride | Hg |
| Methylmercury | Hg |
| Methylmercury_chloride | Hg |
| Oxaliplatin | Pt |
| Seleno-L-methionine | Se |
| Sila-Haloperidol | Si |

**Table S2**. Compounds containing non-standard atoms, for which we used the MCPB.py procedure to generate the General Amber force field parameters.

| Compound | Compound Crystal Code CSD | R-FACTOR (%) | RMSD (Å) |
|---|---|---|---|
| 1,8-Cineole | MOFPAY | 4.66 | 0.02 |
| 1-Butanol | MEXZOG | 3.69 | 0.05 |
| 1-chloro-2,4-dinitrobenzene | BENCLN06 | 3.39 | 1.19 |
| 1-naphthol | NAPHOL01 | 3.74 | 0.05 |
| 2,2'-Dibromobiphenyl | HIQQON | 3.26 | 1.93 |
| 2,3,4-Trichlorophenol | UZOXEN | 3.26 | 0.03 |
| 2,3-Dihydroxynaphthalene | VOGSEP | 3.20 | 0.02 |
| 2,3-Dimethylaniline | QUCRUD | 3.47 | 0.03 |
| 2,6-Di-t-butyl-4-methylphenol | MBPHOL01 | 3.80 | 0.10 |
| 2-I-4-Cl-Aniline | WOHZOK | 2.92 | 0.02 |
| 2-Iodoaniline | RALTOO | 4.80 | 0.03 |
| 3-(2-Methyl-1,3-thiazol-4-yl)ethynylpyridine | OSIYUL | 4.87 | 0.08 |
| 4,4'-Dibromobiphenyl | DBRBIP01 | 3.64 | 0.63 |
| 4-Ethynylbiphenyl | NAMZAC | 4.60 | 0.10 |
| 4-F-aniline | IDAHUR | 3.73 | 0.04 |
| 4-Iodoaniline | EJAYET02 | 2.76 | 0.03 |
| 4-Nitrocatechol | UCIVIP | 2.34 | 0.03 |
| 5-Hydroxymethylfurfural | SUYZUI | 3.63 | 0.86 |
| 7-Ethoxycoumarin | DETFUC | 4.10 | 0.05 |
| Acrolein | AXOSOW | 3.50 | 0.61 |
| Acrylamide | ARCLAM04 | 3.21 | 0.03 |
| Andrographolide | ZZZLUK07 | 3.40 | 0.20 |
| Atrazine | PORJIR03 | 4.42 | 0.50 |
| Biochanin A | IHAHIL | 3.78 | 0.71 |
| Bisphenol F | WIMCOL | 4.64 | 0.20 |
| Busulfan | KADKIJ | 3.97 | 0.66 |
| Caffeine | NIWFEE03 | 4.33 | 0.03 |
| Canrenone | CANREN10 | 4.80 | 0.36 |
| Chloramphenicol | CLMPCL04 | 2.25 | 2.05 |
| Chloroform | CLFORM01 | 3.55 | 0.04 |
| Cilostazol | XOSGUH | 4.10 | 3.06 |
| Ciprofloxacin | UHITOV | 3.70 | 1.09 |
| Clobazam | DAHREJ | 4.30 | 0.06 |
| Coumarin | COUMAR11 | 2.43 | 0.01 |
| Curcumol | COVDUM | 4.60 | 0.04 |
| Cyclohexanone | ZZZWGK01 | 4.62 | 0.02 |
| D-23129 | YETJUF01 | 3.15 | 1.03 |
| Dabrafenib | GICKEM02 | 3.34 | 3.49 |
| Dalcetrapib | IFUWOX04 | 3.91 | 2.73 |
| Dapsone | DAPSUO05 | 3.20 | 0.18 |
| DDT | CPTCET12 | 2.00 | 0.17 |
| Deferiprone | GALDEC | 4.40 | 0.04 |
| Dehydroepiandrosterone | ZOYMOP07 | 2.56 | 0.07 |

| | | | |
|---|---|---|---|
| Diallyldisulfide | RESHAY | 4.98 | 1.57 |
| Diosbulbin-B | HIQHOE | 4.40 | 0.73 |
| Dioxane | CUKCIU02 | 2.40 | 0.01 |
| Diphenylthiourea | ZEYBIO01 | 3.59 | 0.67 |
| Entacapone | OFAZUQ04 | 3.03 | 1.63 |
| Equilenin | QQQAMM01 | 2.92 | 0.05 |
| Ethionamide | ETCYPY | 2.92 | 1.20 |
| Ethyleneoxide | DUFBOV11 | 3.00 | 0.58 |
| Ethylenethiourea | ETTHUR01 | 2.35 | 0.12 |
| Etravirine | LIKTIK | 4.49 | 0.51 |
| Eupatillin | WEHGOG | 4.65 | 1.83 |
| Exemestane | PEKFAN02 | 3.26 | 0.10 |
| Felbamate | CANFED | 3.35 | 2.27 |
| Finasteride | WOLXOK02 | 4.70 | 0.25 |
| Fluconazole | IVUQOF | 4.30 | 1.61 |
| Fluoren-2-amine | MATHIY | 4.30 | 0.04 |
| Flutamide | WEZCOT | 3.40 | 0.33 |
| Geldanamycin | GUTVOJ | 3.83 | 2.66 |
| Ibrutinib | BETXEG | 3.56 | 2.34 |
| Imperatorin | YAGHOE01 | 4.93 | 1.74 |
| Iproniazid | CUXKAK03 | 3.95 | 0.64 |
| Isoniazid | INICAC02 | 2.99 | 1.16 |
| Ketoconazole | KCONAZ | 3.85 | 2.75 |
| Lamotrigine | EFEMUX01 | 2.81 | 0.11 |
| Letrozole | UKAKIA | 4.89 | 0.95 |
| Levetiracetam | OMIVUB09 | 2.64 | 0.64 |
| Macitentan | VEMRAI | 2.82 | 1.72 |
| Medroxyprogesterone acetate | MACXPR10 | 4.20 | 0.39 |
| Menadione | IVEJUO | 3.30 | 0.02 |
| Methoxychlor | MXCLOR | 4.80 | 1.31 |
| Metyrapone | BIHYEW10 | 3.60 | 1.17 |
| Nabumetone | XOCXUI01 | 3.69 | 1.20 |
| Nevirapine | PABHIJ01 | 3.32 | 0.16 |
| Nimesulide | WINWUL02 | 3.40 | 0.95 |
| o-Aminobenzoate Me ester | FASMEV | 4.82 | 1.10 |
| Oltipraz | BIXSOQ10 | 3.40 | 0.29 |
| Ornidazole (R-) | NOBVEF | 3.32 | 0.71 |
| Paracetamol | COTZAN08 | 1.60 | 0.16 |
| Pentachlorophenol | PCPHOL01 | 1.09 | 0.04 |
| Phenylthiourea | QQQDDM01 | 3.70 | 0.14 |
| Pterosin A, (2S)- | UMAZEP | 3.12 | 0.51 |
| Rivaroxaban | LEMSOO01 | 2.62 | 1.56 |
| Rofecoxib | CAXMUJ | 4.20 | 1.42 |
| Rotenone | PAXWAN | 3.98 | 0.80 |
| Simvastatin | EJEQAL04 | 4.76 | 1.28 |
| Sorafenib | AKENOU | 3.68 | 1.97 |

| | | | |
|---|---|---|---|
| Spironolactone | ATPRCL01 | 4.70 | 0.38 |
| Sterigmatocystin | STERIH10 | 3.90 | 0.34 |
| Styrene | ZZZTKA02 | 3.76 | 0.03 |
| Sulfamethoxazole | SLFNMB09 | 2.80 | 1.34 |
| Sulfapyridine | BEWKUJ04 | 4.10 | 1.69 |
| Tetrafluoroethene | GAFLIJ | 2.10 | 0.01 |
| Thalidominde, (S-) | THALID12 | 4.67 | 1.60 |
| Thioacetamide | THACEM01 | 2.60 | 0.02 |
| Tioconazole, (R)- | ROBHAT | 2.84 | 2.59 |
| Tolbutamide | ZZZPUS19 | 2.66 | 1.02 |
| Zibotentan | OCAGAB | 4.67 | 0.73 |
| Average | - | - | **0.76** |

**Table S3** RMSD values (Å) calculated on the heavy atoms between CSD and DFT optimised structures, together with CSD code and R-factor (%). A sample of 100 molecules has been considered.

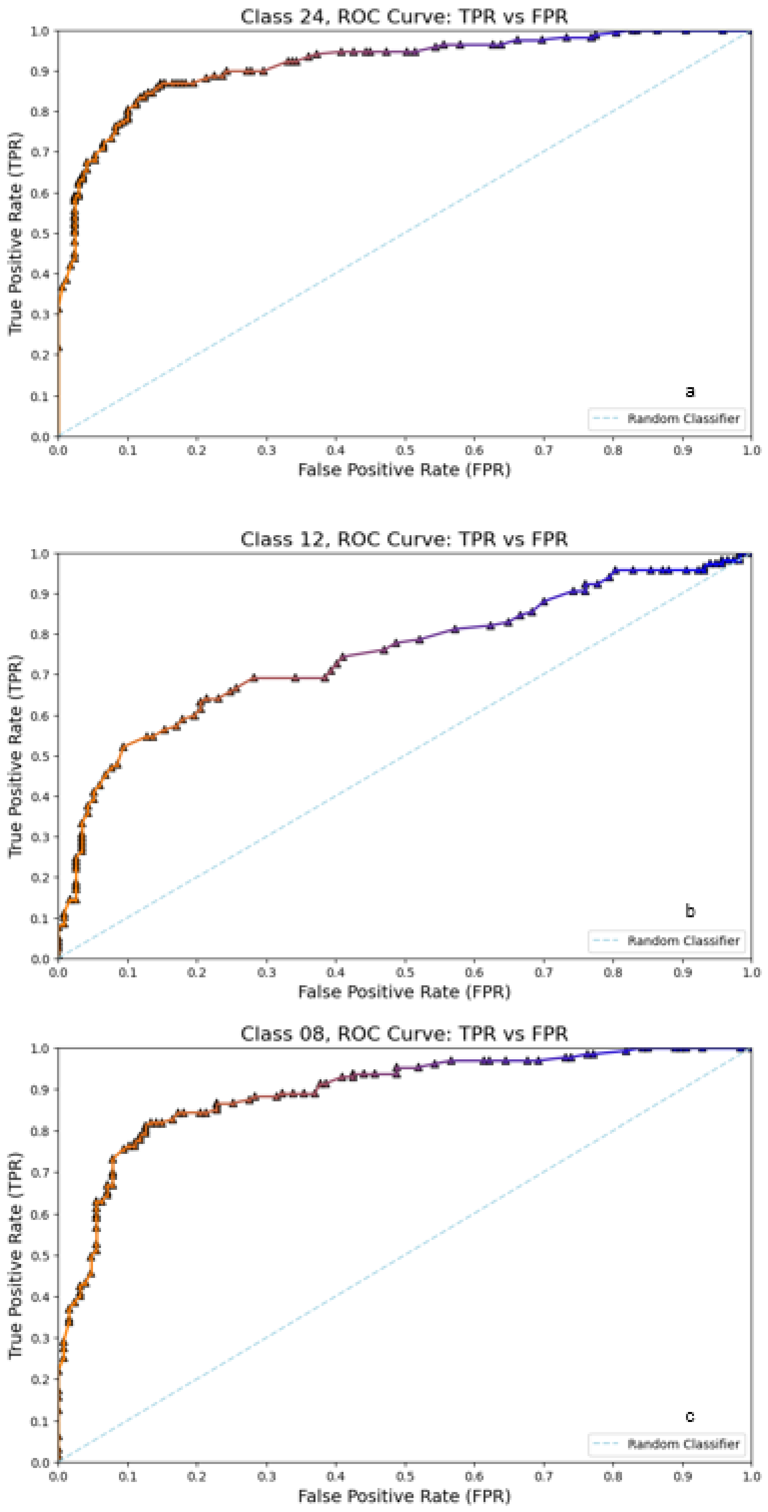


**Figure S1.** ROC Curves (True Positive Rate vs. False Positive Rate) for the models evaluated with 10-fold cross-validation, after features selection, for the three classes. Panel (a) displays the ROC curve for Class 24 (ROC Area = 0.92), panel (b) shows the ROC curve for Class 12 (ROC Area = 0.75), and panel (c) displays the ROC curve for Class 08 (ROC Area = 0.89). The dashed lightblue line represents a random classifier (ROC Area = 0.5).
